# The Use of Aspirin to Reduce the Risk of Thrombotic Events in Patients With End-Stage Renal Disease: Protocol for a Randomized Controlled Trial

**DOI:** 10.2196/10516

**Published:** 2018-08-09

**Authors:** Tiago Lemos Cerqueira, Armando Fartolino Guerrero, Clara Krystal Pérez Fermin, Ricardo Wang, Evelin Elfriede Balbino, Janis L Breeze, Paola Gonzalez Mego, Daniele Argentina Silva, Walid Ezzeldin Omer, Nathalie Monique Vandevelde

**Affiliations:** ^1^ Betim Nephrology Unit Associaçao Evangélica Beneficente de Minas Gerais Belo Horizonte Brazil; ^2^ Harvard TH Chan School of Public Health Harvard University Boston, MA United States; ^3^ Dresden International University Dresden Germany; ^4^ Family Health Strategy São Paulo Municipal Health Department São Paulo Brazil; ^5^ Hematology-Oncology Unit Hospital Infantil Regional Universitario Dr Arturo Grullón Santiago De Los Caballeros Dominican Republic; ^6^ Cardiology Department Santa Casa de Belo Horizonte, Unimed Belo Horizonte Belo Horizonte Brazil; ^7^ Agência Nacional de Vigilância Sanitária Brasilia Brazil; ^8^ Tufts Clinical and Translational Science Institute Tufts University Boston, MA United States; ^9^ Institute for Clinical Research and Health Policy Studies Tufts Medical Center Tufts University Boston, MA United States; ^10^ Suiza Laboratory Lima Peru; ^11^ Faculdades Souza Marques Rio de Janeiro Brazil; ^12^ Hearing & Balance Unit Hamad Medical Corporation Doha Qatar; ^13^ Department of Quality of Medical Laboratories Scientific Institute of Public Health Brussels Belgium

**Keywords:** randomized controlled trial, end stage renal disease, kidney failure, chronic, aspirin, prophylaxis, thrombosis, drug safety, bleeding, platelet activation, diabetes mellitus, type 2

## Abstract

**Background:**

End-stage renal disease (ESRD) is the last stage of chronic kidney disease, mainly caused by type 2 diabetes mellitus and characterized by an increased mortality risk related to cardiovascular disease. Low-dose aspirin (acetylsalicylic acid or ASA) seems to effectively prevent cardiovascular events in patients with ESRD. However, the number of interventional studies in this population remains limited and the mechanisms of aspirin-related bleeding remain poorly understood. Aspirin’s efficacy and safety may be modified by the presence of type 2 diabetes mellitus or platelet hyperreactivity.

**Objective:**

The overall objective of this protocol is to (1) evaluate aspirin’s safety and efficacy in reducing the risk of thrombotic events in patients with ESRD on hemodialysis and (2) examine whether aspirin’s efficacy is modified by the presence of type 2 diabetes mellitus or platelet hyperreactivity. Specifically, the primary objective is to compare the 12-month rate of any thrombotic event (cardiac death, nonfatal myocardial infarction, nonfatal stroke, arteriovenous fistula thrombosis) and Thrombolysis in Myocardial Infarction (TIMI) major bleeding in patients treated with aspirin compared to those on placebo. Secondary objectives are to test for effect modification of treatment by the presence of type 2 diabetes mellitus or platelet hyperreactivity and compare the rate of TIMI minor bleeding between treatment groups.

**Methods:**

We developed a protocol for a phase 2 randomized, single-center, placebo-controlled, triple-blind, superiority clinical trial to assess the prophylactic efficacy and safety of aspirin in patients with ESRD and on hemodialysis. It follows the ethical principles of the Declaration of Helsinki of the World Medical Association. A total of 342 participants would be enrolled over 12 months at a large dialysis center. Patients will be randomized in a 1:1 ratio and stratified by presence of type 2 diabetes mellitus and platelet hyperreactivity to receive either oral aspirin (100 mg/d) or placebo for a treatment period of 12 months. An intention-to-treat statistical analysis will be performed.

**Results:**

The randomized clinical trial will be performed after approval by the ethical committee of the participating center and registration at ClinicalTrials.gov.

**Conclusions:**

We provide a protocol for a randomized controlled trial to evaluate the safety and efficacy of treatment with aspirin to reduce the risk of thrombotic events. In addition, such a study would further our understanding of the mechanism of aspirin-related bleeding and help identify subgroups of best-responders and patients with a higher risk of adverse events.

**Registered Report Identifier:**

RR1-10.2196/10516

**Protocol**

## Introduction

### Background and Rationale

Chronic kidney disease (CKD) is a pathology defined by a decrease in glomerular filtration rate (GFR) below 60 mL/min/1.73 m^2^ or by the presence of kidney damage for at least 3 months [1]. It affects 200 million people worldwide, and the main risk factor for this disease is diabetes mellitus [2-4]. The life expectancy of CKD patients decreases with the severity of kidney impairment [5]. The most severe stage is end-stage renal disease (ESRD) and refers to patients undergoing renal replacement therapy (RRT) or for whom the GFR is lower than 15 mL/min/1.73 m^2^ [1]. The high risk of mortality observed in patients with ESRD is mainly due to cardiovascular events, the risks of which are 10- to 20-fold higher in these patients compared to non-CKD subjects and are significantly increased by the presence of diabetes mellitus [4,6,7]. In order to prevent cardiovascular events, patients with ESRD receive anticoagulant or antiplatelet therapy [8,9].

The benefits of low doses (75 to 100 mg/d) of aspirin (acetylsalicylic acid or ASA), an antiplatelet agent, as prophylactic drug for some specific types of cardiovascular events (atherosclerotic and ischemic events) in CKD and patients with ESRD have been reported in several studies. For instance, the impact of low-dose aspirin (75 mg/d) versus placebo on the risk of cardiovascular events was reported in an interventional study of 3619 CKD patients with hypertension (eGFR <60 mL/min/1.73 m^2^ at enrollment) [10]. The hazard ratio (HR) of cardiovascular events decreased by 15% (HR 0.85, 95% CI 0.61 to 1.17; *P*=.03) in patients with an eGFR of 45 to 59 mL/min/1.73 m^2^ and 66% for patients with eGFR <45 mL/min/1.73 m^2^ (HR 0.34, 95% CI 0.17 to 0.67; *P*<.05) [10]. Among secondary end points, a 36% reduction in the rate of myocardial infarction (HR 0.64, 95% CI 0.39 to 1.03; *P*=.08) was observed in patients with an eGFR of 45 to 59 mL/min/1.73 m^2^, and subjects with eGFR <45 mL/min/1.73 m^2^ had a rate reduction of 69% (HR 0.31, 95% CI 0.11 to 0.85; *P*<.05). Stroke, cardiovascular mortality, and total mortality were also reduced by 50% to 80% in patients with eGFR <45 mL/min/1.73 m^2^ receiving aspirin compared to placebo [10]. A systematic review of 2572 randomized controlled trials, meta-analyses, and systematic reviews (27 retained) reported that aspirin was associated with a 6% reduction in the relative risk (RR) for all-cause mortality (RR 0.94, 95% CI 0.88 to 1.00), 10% reduction in major cardiovascular events (RR 0.90, 95% CI 0.85 to 0.96), and 15% reduction in total coronary heart disease (RR 0.85, 95% CI 0.69 to 1.06) [11]. Last, a large case-control observational study performed on stroke patients with ESRD undergoing dialysis between 1998 and 2006 and exposed (n=763) or not (n=666) to aspirin (80 to 325 mg/d) showed significantly lower rates of all-cause mortality (HR 0.671, 95% CI 0.580 to 0.777; *P*<.001) and readmission to hospital for ischemic stroke (HR 0.715, 95% CI 0.580 to 0.882; *P*=.002) in the group receiving aspirin versus placebo, without any significant increase of risk of bleeding (*P*=.29) [12].

However, there is a lack of information about the prophylactic efficacy of aspirin for all types of thrombotic events that patients with ESRD may develop. There is also a gap of knowledge concerning the safety profile of aspirin in patients with ESRD. Concerning the risk of aspirin-related bleeding, there is some discrepancy between the results of observational and interventional studies, as an increased risk of bleeding has been reported in some observational studies [13,14]. However, interventional studies of patients with ESRD have found that low doses of aspirin are not associated with an increased risk of major bleeding in dialysis patients, despite an apparent increased risk of minor bleeding (eg, gastrointestinal bleeding) [10,15]. This discrepancy highlights the fact that the mechanism of aspirin-related bleeding events is not yet fully understood. Some authors have suggested that the prophylactic efficacy of aspirin and risk of bleeding related to this drug may be influenced by a phenomenon of platelet hyperreactivity [16]. However, further research is necessary to establish the real impact of platelet hyperreactivity on aspirin’s safety profile in patients with ESRD.

### Objectives

The primary and secondary objectives of this study are to evaluate aspirin’s prophylactic efficacy and safety in patients with ESRD. This will include (1) the assessment of aspirin prophylactic efficacy for all types of thrombotic events that patients with ESRD may develop, namely nonfatal stroke, nonfatal myocardial infarction, arteriovenous fistula thrombosis, and cardiac mortality, and (2) the assessment of aspirin-related major bleeding events using Thrombolysis in Myocardial Infarction (TIMI) criteria [17-19]. The study’s secondary objectives are to test for effect modification of treatment by the presence of type 2 diabetes mellitus or platelet hyperreactivity and compare the rate of TIMI minor bleeding between treatment groups.

Our hypothesis is that aspirin is superior to placebo as prophylactic therapy for thrombotic events in patients with ESRD on hemodialysis without increasing the risk of major bleeding.

## Methods

### Participants, Interventions, and Outcomes

#### Study Design

**Figure 1:**
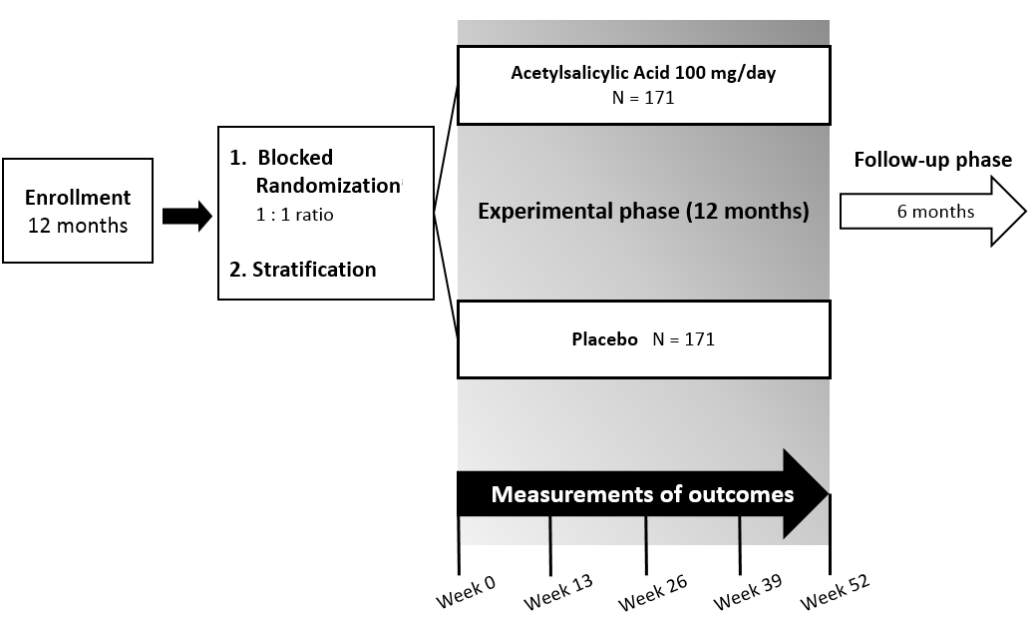
Illustration of the study design. Participants will be randomized in block sizes of 4, 6, and 8. Stratification factors: type 2 diabetes; platelet hyperreactivity.

We will perform a phase 2 randomized, single-center, placebo-controlled, triple-blind, superiority clinical trial with 1:1 allocation to receive either 100 mg of aspirin per day or placebo by mouth for 12 months. Randomization will be stratified based on 2 baseline characteristics: (1) the presence versus absence of type 2 diabetes mellitus and (2) the presence versus absence of platelet hyperreactivity. The study will start following approval of the Institutional Review Board (IRB) and will follow the ethical principles of the Declaration of Helsinki of the World Medical Association. The study design is illustrated in Figure 1.

#### Study Setting

The trial involves patients on chronic intermittent hemodialysis and will be conducted in a large dialysis center (ie, with ≥2000 patients on RRT).

#### Eligibility Criteria

The study population consists of men or women with ESRD who are at least 18 years of age and have started chronic intermittent hemodialysis in the previous 3 months.

Exclusion criteria include any contraindications to ASA, concurrent treatment with anticoagulants or platelet aggregation inhibitors, and pregnancy or lactation. Patients with life-threatening conditions other than renal or vascular disease will also be excluded from the trial: all types of cancer, liver disease, AIDS, or severe lung disease. ESRD due to glomerulopathy has a different pattern of mortality [20], which precludes its inclusion in this trial. Patients on other modalities of RRT will also be excluded.

#### Interventions

The intervention is an enteric-coated oral pill containing 100 mg of ASA, administered once daily, after lunch, over 12 months. The comparator will be a placebo tablet with the same characteristics as the active pills. Both arms will have the same administration schedule and will start treatment the day after randomization (day 0).

#### Adherence

At every hemodialysis session, participants will receive training about the importance of taking the drug as prescribed by viewing a 10-minute educational video about medication compliance on a digital tablet.

Study patients will have monthly visits with the study team to check on treatment adherence and answer any questions. During these visits, patient compliance with treatment will be assessed by direct questioning and by counting returned tablets provided on a monthly basis. Good adherence will be defined as taking at least 80% of the prescribed daily dose and attending 100% of the visits. If a patient withdraws from the trial, the study coordinator will contact the participant to find out the reasons for withdrawal.

#### Outcomes

The primary outcome is the incidence of a composite event including all of the following: cardiac death, nonfatal myocardial infarction, nonfatal stroke, arteriovenous fistula thrombosis, and TIMI major bleeding [19] by 12 months of treatment. The secondary outcomes are effect modification of treatment efficacy by the presence of type 2 diabetes mellitus or platelet hyperreactivity and the rate of TIMI minor bleeding between treatment groups.

According to the TIMI criteria, major bleeding is defined as the presence of any intracranial bleeding (excluding microhemorrhages <10 mm evident only on gradient-echo magnetic resonance imaging), fatal bleeding (bleeding that directly results in death within 7 days), or clinically overt signs of hemorrhage associated with a drop in hemoglobin of ≥5 g/dL or a ≥15% absolute decrease in hematocrit [19]. Minor bleeding is defined as clinically overt with a hemoglobin drop of 3 to 5 g/dL [19].

The profile of platelet reactivity will be determined by multiple electrode aggregometry using a Multiplate analyzer (Dynabyte GmBH). This method was chosen because it currently is the most efficient assay used to assess platelet reactivity in humans [21]. The Multiplate will measure platelet activity (defined as aggregation capability after activation with adenosine diphosphate) as the area under the curve reported in area units multiplied by time (AU×min). Platelet hyperreactivity is defined based on the manufacturer’s recommendations and published data, with a cutoff of 50 AU×min for adenosine diphosphate–induced aggregation [22].

#### Outcomes Assessment

A blinded multidisciplinary team composed of a nephrologist, cardiologist, and nurse will follow participants and review medical records and adjudicate outcomes and end points in the study.

A medical appointment will be scheduled for weeks 0, 13, 26, 39, and 52, where the multidisciplinary team will assess the primary and secondary outcomes and report them to the Data Coordinating Center (DCC). At each visit, a venous blood sample (5 mL) will be collected in order to measure hematocrit, hemoglobin, and platelet hyperreactivity.

After the end of the treatment phase of the trial, participants will be followed for an additional 6 months to assess further adverse events. For that purpose, the multidisciplinary team will contact patients monthly during their hemodialysis sessions. The total length of the study will be 30 months.

#### Study Variables

The outcome variables are the incidence of cardiac death, nonfatal myocardial infarction, nonfatal stroke, and arteriovenous fistula thrombosis, as well as the presence or absence of bleeding and its severity based on TIMI criteria [19]. The treatment variables are the exposure to the intervention (aspirin 100 mg/d or placebo). The stratification variables are the presence versus absence of type 2 diabetes mellitus and the presence versus absence of platelet hyperreactivity.

#### Participant Timeline

The schedule and procedures during patient visits to the participating center (recruitment, hemodialysis sessions, and monthly assessment of outcomes) are summarized in Table 1.

#### Sample Size

Sample size was calculated based on estimates for the rate of events in the primary composite outcome over 1 year: TIMI major bleeding (placebo 1% vs aspirin 2.5% [15]), cardiovascular events (placebo 3.0% vs aspirin 2.5% [10]), and fistula thrombosis (placebo 19% vs aspirin 8% [23]), for a total 1-year event rate of 23% in the placebo group and 13% in the aspirin group. A sample size of 318 patients (159 per arm) provides 80% power to detect a difference between groups of this magnitude (corresponding to an HR of 0.53) using a 2-sided log-rank test and alpha=.05 (Power Analysis and Sample Size Software version 14, NCSS LLC). The sample size for the trial was increased to 342 patients (171 per arm) to account for estimated attrition of 10%.

#### Recruitment

The estimated hemodialysis admission rate at a large dialysis center is 50 patients per month. Therefore, we expect to include 8 to 10 patients every week, completing our recruitment goal in 9 months. We propose a recruitment period of 12 months in case enrollment is slower than expected.

Eligible patients will be identified using medical records, clinician invitation letters, and internal flyers posted in patient areas. The study team will approach eligible patients to invite them to participate in the trial. All aspects of the study will be explained and all participants who agree to participate must provide written informed consent. One venous blood sample will be collected prior to randomization in order to assess platelet reactivity profile, a stratification factor for randomization.

**Table 1:** Schedule and procedures during patient visits to the participating center.

Procedure	Enrollment phase (12 months)	Experimental phase (12 months)					Follow-up (6 months)
	Preliminary visit (369 to 3 days before the experimental phase)	First medical visit (day 0)	Hemodialysis sessions (approximately every 3 days)	Every 30 days	Weeks 13, 26, 39 and 52	Hemodialysis sessions (every 30 days)	
Informed consent form	X^a^						
Physical exam/medical history and medication review	X						
Inclusion/Exclusion	X						
Collection of a blood sample^b^		X			X		
Randomization		X					
Patients’ meeting with the study team and oral training about compliance to treatment		X		X			
Provision of the necessary number of pills (on a monthly basis)		X		X			
Patients’ training focused on compliance (short educative video)			X				
Counting of returned pills				X			
Blinding assessment (questionnaire)				X			
Measurement of primary and secondary outcomes^c^ during consultations with a nephrologist and a cardiologist		X			X		
Assessment of later bleeding adverse events						X	

^a^X indicates the time at which each procedure will occur.

^b^Assessment of the hematocrit, hemoglobin concentration, and platelet hyperreactivity profile.

^c^Number of events for cardiac death, nonfatal myocardial infarction, nonfatal stroke, arteriovenous fistula thrombosis, and assessment of major and minor bleeding using the Thrombolysis in Myocardial Infarction scale.

### Assignment of Interventions

#### Sequence Generation

Patients will be allocated to 1 of the 2 study groups based on a computed-generated blocked randomization, with stratification by 2 factors (type 2 diabetes mellitus and platelet hyperreactivity). For that purpose, randomly permuted blocks of sizes 4, 6, and 8 will be used in order to maintain the integrity of the randomization and blinding [24]. The software used to generate the sequence is available at randomization.com, and the study pharmacists will coordinate the randomization, treatment assignment, and delivery of study medication [25].

The research pharmacists will determine the study allocation and randomization and will be the only individuals to know the identity of the drugs delivered.

#### Blinding

The trial is triple-blinded to the treatment allocation: participants, study clinicians, and staff, as well as data analysts, will not know participant treatment assignments. Blinding will be assured by the use of a placebo comparator, which will be identical in look and taste to the active drug and will last until the data are analyzed.

A questionnaire will be used to assess the effectiveness of the participant’s blinding. This will be performed according to the methodology proposed by Rees and collaborators [26]. Every 2 weeks, participants will be asked to complete a survey about the intervention they think have received (active intervention, placebo, or unknown) and their level of certainty on a Likert scale. The accuracy of their answers will be evaluated using the Howard index, and differences in beliefs between 2 successive questionnaires will be assessed using the Fisher exact test. Participants’ beliefs about their treatment assignment are considered consistent if all responses are sequentially identical over time, except one change of opinion that may be explained by a lack of blinding [26].

#### Emergency Unblinding

In exceptional circumstances, unblinding may happen if knowledge of the actual treatment is essential for further management of the patient. In case of severe adverse events, investigators will discuss unblinding within 24 hours with a medical advisor from the Data and Safety Monitoring Board who is not involved with the trial. Unblinding will take place by the research pharmacy immediately after the decision is made.

### Data Collection, Management Analysis, and Monitoring

#### Data Collection

We intend to minimize missing data by having monthly visits with patients. An adherence check will be done by counting returned tablets as previously stated.

Regarding data collection and storage, the study team will be trained to oversee all key aspects of the protocol: (1) methodology, forms, and tools that must be used for the collection, entry, monitoring, and editing of data; (2) appropriated methods to communicate among investigators and between investigators and participants; and (3) importance of reporting data as close to real time as possible during the course of the study.

#### Data Management

For quality control, patient records (source documents) will be stored at the site, and the original data will be shared with the DCC. It is the responsibility of the investigator to keep, maintain, and provide the documents audited by IRB, sponsor, National Institutes of Health, US Food and Drug Administration, or other local regulatory agencies when necessary.

After the collection of study data, patient identification will be encoded, and only the investigator will have access to this information, in accordance with the Good Clinical Practices and the Declaration of Helsinki regarding confidentiality [27].

The collected data will be entered electronically in a Research Electronic Data Capture management system. This database is a cloud-based system, and it will have a backup in a hard disk in the DCC. The dataset will be encrypted in order to guarantee data safety and confidentiality.

#### Statistical Methods

Statistical analysis will be performed including all randomized patients according to their assigned treatment group (ie, intention-to-treat). The software used will be Stata 14 (StataCorp LLC). All testing will be 2-sided with statistical significance defined as *P*<.05. The primary composite outcome (first time-to-event of thrombotic events and TIMI major bleeding) will be analyzed with Kaplan-Meier curves and a log-rank test to detect difference between the groups. Cox proportional hazards regression may be used to adjust for relevant covariates, if appropriate (eg, for any unbalanced baseline characteristics that may occur by chance). Similarly, secondary outcomes will be analyzed with Kaplan-Meier curves and log-rank tests for all events. For the secondary objectives, interaction terms will be included in the Cox models above to test for interaction between treatment status and (1) the presence of type 2 diabetes and (2) platelet hyperreactivity. The proportion of patients with TIMI minor bleeding in each group will be compared with a chi-square test. If necessary, multiple imputation will be used for missing data.

#### Data Monitoring

A Data and Safety Monitoring Board consisting of an independent nephrologist and cardiologist (adverse events may happen mainly in those fields) and statistician is planned to oversee the trial. Based on federal regulations, an ethicist may be included. According to our inclusion and exclusion criteria, no vulnerable population is targeted.

### Ethics and Dissemination

#### Institutional Review Board Submission

Prior to recruitment of study subjects, the full study protocol will be submitted to the local research ethics committee and IRB for evaluation and approval.

#### Registration

The trial will be registered at ClinicalTrials.gov.

## Results

This is a protocol for a randomized clinical trial. It must be submitted to an ethical committee and registered at ClinicalTrials.gov before we can specify dates of data collection or the beginning of the study. These practical aspects also depend on the decisions of the center where the trial would be performed.

## Discussion

### Summary

Finding a safe preventive measure for thrombotic events for patients with ESRD on hemodialysis is of utmost importance, as it would reduce the number of fistula thrombosis and cardiovascular events and have a direct impact on morbidity and mortality rates. This clinical trial will provide data on the safety of antiplatelet blockade with aspirin, which is a possible preventive measure for thrombosis, and assess the impact of the intervention in hemodialysis patients. In addition, it will yield essential information to foster further interventional trials and may help revise international guidelines for the prevention of thrombotic disease in patients with ESRD on hemodialysis.

### Strengths and Limitations

The main strength of the trial is its study design, which includes allocation concealment, randomization, and triple-blinding in order to reduce possible bias. In addition, stratification will balance 2 variables that are strongly associated with the outcome, type 2 diabetes mellitus and platelet hyperreactivity, and will allow us to explore whether patients with these characteristics differentially respond to aspirin therapy. Overall, the study protocol is feasible for both investigators and patients, as study enrollment and subsequent visits will take place during or following the patients’ standard hemodialysis sessions.

Potential limitations of the study protocol include the difficulty in interpreting a composite outcome. The use of a composite primary outcome is supported by the fact that major bleeding rates are very rare, requiring a large sample size and lengthy study duration that would render the trial unfeasible. We address this issue by adding efficacy outcomes related to thrombosis to major bleeding outcomes. In order to clarify interpretation of the primary composite outcome, they are individualized in the secondary analysis. Regarding the study population, peritoneal dialysis patients will not be included as most do not have arteriovenous fistula and, hence, are not at risk for fistula thrombosis. Furthermore, glomerular disease patients who developed ESRD will not be included, as they show a different pattern of morbidity and mortality [20]. Another limitation is the possibility that we do not meet the planned recruitment time, as it will affect study power and validity. For that matter, we allow 12 more weeks for recruitment than initially planned. Last, despite the simplicity of drug administration, adherence is always a potential problem, which will be dealt with by identification of nonadherent patients and systematic training.

### Conclusion

The study protocol will provide essential evidence to foster further clinical research on preventive measures of thrombotic events in hemodialysis patients. Future research is needed to provide information about the impact of preventive antiplatelet blockage on mortality and thrombotic events in these patients. Moreover, the study of potential biomarkers to identify patients who would benefit the most from the intervention is also required and may have a direct effect on drug prescription and control of adverse events. Therefore, identifying the potential safety and effectiveness of aspirin will improve morbidity and mortality, lowering the burden of such a severe disease for these patients and giving them a chance for a better and longer life.

## Abbreviations

ASA: acetylsalicylic acid
CKD: chronic kidney disease
DCC: Data Coordinating Center
ESRD: end-stage renal disease
GFR: glomerular filtration rate
HR: hazard ratio
IRB: Institutional Review Board
RR: relative risk
RRT: renal replacement therapy
TIMI: Thrombolysis In Myocardial Infarction
